# The Principles and Practice of Dental Surgery

**Published:** 1845-03

**Authors:** 


### Bibliographical Notices.

*The Principles and Practice of Dental Surgery.* By CHAPIN A. HARRIS, M. D., D. D. S., Professor of Practical Dentistry, and Dental Pathology, in the Baltimore College of Dental Surgery. Member of the Medico-Chirurgical Faculty of Maryland, &c. &c. Second Edition; revised, modified, and greatly enlarged: Illustrated by sixty-nine wood engravings. Philadelphia: LINDSAY & BLAKISTON, 1845—pp. 600, 8vo.

THE above work came to hand only a day or two since, and we greatly regret our inability to do it justice, in the limited time, (the forthcoming No. of the Journal, being already due,) we have to prepare any notice, or review of this very admirable and extensive treatise. Our task is, however, much abridged by the able review of the *first* edition, by SOLYMAN BROWN, Vol. 1, No. 1, page 8, of the American Journal of Dental Science:—leaving it for us only to notice some of the many and very great additions, which the second edition contains. We can, indeed, in no way better express, in general terms, our own view of either edition, and especially of the latter, than by quoting from the article alluded to, from the able pen of Dr. Brown.

He thus remarks—(Dental Journal, Vol. 1, No. 1, page 8,) "The members of the profession, and students in particular, have long felt the necessity of a treatise on dentistry, in all its branches, Medical, Surgical and Mechanical, which should at once be comprehensive in its scope, practical in its detail, correct in its science, and beautiful in typographical execution. This desideratum is now supplied in a manner highly creditable to the accomplished author, and peculiarly acceptable to the profession at large. * * * Dr. Harris has proved himself so well qualified for his task, that it may be safely asserted, that no one volume in the English language, contains an equal amount of correct and valuable information for the use of the student in dentistry. * * There are manifested also in Dr. Harris' volume, an originality of thought, and an independence of opinion, equally calculated to elicit truth and expose error.

"But there is still another point of view in which this treatise should be regarded, by all who are either operators or subjects of dental practice. It is this:—The author has so well acquitted himself, that his work is calculated to give dignity and importance to the subject of which it treats, evincing that the writer is one of those high-minded men who impart respectability to the calling which they pursue, rather than increase their own importance by its exercise."

But as the first edition has long been before the profession, and its merits fully appreciated and *acknowledged,* we may pass directly to notice some of the leading improvements introduced into the second edition—improvements well worthy one who has long been signalized for his vigor of thought, practical sense, and boldness of enterprise. This edition is divided into six parts.

The first, comprehends the Anatomy and Physiology of the Mouth.

The second, The Physical Characteristics of the Teeth, Gums, Salivary Calculus, &c.

The third, Diseases of the Teeth.

The fourth, Salivary Calculus—Diseases of the Gums, &c.

The fifth, Diseases of the Maxillary Sinus.

The sixth, Mechanical Dentistry.

In comparing this edition with the first, it is proper to observe, that the parts, first, second and fifth, are additions, as new matter, to the first edition. We will first notice the importance of the subjects included in the first part, and some of the reasons why they should have a place in an *elementary* work on dentistry. It has long been not only a source of regret, but of great mortification, to every honorable and intelligent member of the dental profession, to see such profound ignorance on the part of so many of its practitioners, in respect to the *principles* upon which such practice should be based. Thousands, we venture to say, have plodded along in it, for years, without knowing any more of the anatomical structure of the mouth, and its appendages, than could be learned from an examination of the surface. Not only ignorant of the various tissues and their arrangements, but still more so, in respect to the laws applicable to each, and their modifications arising from their connection with other parts of the system. Nor are we wholly at a loss to account for this very common, yet nevertheless disgusting exhibition of ignorance, as connected with the dental profession, when we consider how perfectly destitute most of our works on dentistry are, of any information in relation either to anatomical or physiological facts, which must ever constitute the basis of correct practice. The truth is, that dental science, (at least so far as most of our dental works are concerned,) has commenced where medical science ends. In other words most of our dental authors have apparently gone upon the supposition that every dental student had already become familiar, not only with the ***structure***, but all the ***laws***, pertaining to the human system, both in health and disease. Upon the very first page, allusions are not unfrequently made to a greater number of structures and functions, than could be comprehended in months of the closest application—and what is still worse, they furnish no explanation which will in the least serve to enlighten him.

Judging from their *works*, we should be led to imagine that such authors had adopted one of the three following conclusions, viz. either that a knowledge of structure and function are not essential, as relates to those organs which are to be the subjects of treatment, or that the student has previously acquired such knowledge, which would be far from the fact, as applied to thousands who are about to commence dentistry, or, (which is still more absurd,) that the *allusion*, necessarily brings to the mind of the student an intuitive knowledge and comprehension of the subjects, thus alluded to. Feeling that it would be by no means safe, to adopt either of these conclusions, but, on the contrary, going upon the supposition, that such knowledge *is* necessary as the basis of all *safe* dental practice, we inquire what must be the effect upon the mind of the beginner who had adopted this belief. The conviction strikes him at once that he must seek some other source of information than dental works, to pilot him out of the mist, by which he finds himself surrounded. He is directed, perhaps, to a medical library, but the imposing front which it presents, with its thousand volumes, generally either drives him to despair, or induces the resolve, that he will make the most of his dental works, "skipping the hard words" which he cannot comprehend. We can then, but hail the appearance of any new work upon our favorite science, which promises to obviate this great defect in the productions of most authors on dental science, with a most cordial welcome.

Dr. Harris has introduced into his second edition so much of anatomy and physiology, illustrated by very neat and appropriate cuts, as that the dental student may get a very good idea of the structures and functions of those organs he is to deal with, and this too, in a form at once the most simple and concise.

And it should be observed, that to accomplish this very desirable end, is no easy task, especially to bring it within the limited space which this topic occupies in the above treatise, and one which has never been effected by any other American author. It cannot be expected that a very comprehensive knowledge of anatomy and physiology could be obtained, even of those organs which may be regarded strictly dental, from any treatise upon these subjects, so much abridged as it must be, as a part of a dental work, yet as narrow as are the limits in the work under consideration, much more valuable information may be derived from it, relating to these subjects, than is ever obtained by one dentist of a hundred, who had not previously studied medical science. We are well pleased also with the arrangment of subjects. It seems to us as natural and easy as could be adopted. Principles are first stated and elucidated, thus making, what would otherwise be arbitrary rules of practice, *deductions,* from clearly illustrated and well established laws of the human economy. The author's views in relation to the importance of studying the *connection* of parts, that we may fully comprehend any one, entirely accords with our own, and we believe it is the only view which will lead to correct practice. From the following quotation upon this point we may see something of the views which he advocates, and which he has admirably succeeded in carrying out in the second edition of his work. Under the head of "*Anatomical* Relations of the Mouth," he thus remarks :

"The mouth has many interesting anatomical relations with the rest of the body, a few of which it may be well to mention.

"By means of its lining mucous membrane, it is connected through similar continuity of structure with the stomach and the whole of the intestinal canal, liver, &c. &c.

"Disease still further establishes this structural relation. Inflammation, ulceration, or any other anatomical change in the stomach or intestines is felt and reported on the tongue, gums and other parts of the mouth, showing the sympathy and the close anatomical relationship of these several parts. The mouth is also connected by the same mucous membrane with the organs of respiration, by being continued down into the larynx, trachea and bronchia.

"By the fifth pair of nerves, the mouth, and especially the dental apparatus, has a most important relation with the brain, nervous system, and all parts dependent on them.

"Simple irritation from teething has frequently thrown children into convulsions—and in adults, tooth-ache often creates extreme irritability of the whole nervous system. But it is not necessary to dwell here on the morbid sympathies of the mouth with other parts of the body, as it is the intention of the author to refer more particularly to the subject, in a subsequent part of the work. It will be well, however, to mention in this place, that there is a general anatomical relation of the mouth with the rest of the body, by means of the blood-vessels and cellular tissue.

"These latter are the most pervading general elements of the body; they are found every where connecting, and binding together, all the organs in one great and common family, and in this manner the mouth is related to and forms an essential link in this natural chain." (pp. 93, 94.)

Again, under the head of "Physiological Relations of the Mouth," he remarks:

"The mouth has been shown to consist, not only of a great variety of parts, but also, that it has an equally great variety of functions.

"The functions of the mouth have been stated to be those of prehension, mastication, insalivation and deglutition.

"These functions, it has been seen, are all closely related the one with the other, and mutually dependent—and how beautiful is the harmony of action as well as its regular and orderly succession. We see in the first place, the prehensile instruments laying hold of and introducing the food into the mouth—then the organs of mastication, the teeth and upper and lower jaw bones, put into operation by the temporal, masseter and pterygoid muscles, grind it down into minute portions, which, at the same time, are formed into a bolus by being mixed with the salivary fluids, furnished by the parotid, sub-maxillary, and sub-lingual glands; then it is taken by the organs of deglutition, to wit: the tongue, palate and pharynx, and passed by these into the œsophagus, to be thence conducted into the stomach—thus demonstrating the harmony of relation among the several functions belonging to the mouth.

"But the functional relations of the mouth are not more confined to itself than its structural relation, the one is equally commensurate with the other; and as the structure of the mouth has been shown to be continuous with, and to extend to the most extreme parts of the body, so we find that the functions of the mouth equally involve all the great, general and leading functions of the body.

"For example, if the primary stages of digestion be impaired or improperly performed in the mouth, the whole process of digestion must also be necessarily imperfect, the stomach will form bad chyme, the intestines bad chyle, and the impure fluid will go to the heart and lungs, where bad blood will be formed, and as a necessary consequence, the functions of circulation, respiration, and inervation also implicated, and in this way we see, that by disturbing only one link, and at once, the brotherhood of the great functional chain becomes broken.

"Again, the mouth is intimately related with the intellectual functions, as for instance that of speech. Who does not know that when any of the teeth are wanting, the palate cleft, or there is a hare-lip, how much the speech is impaired?

"And so with all the other functions of the body, the relation between them and the mouth, and the mutual dependence of each on the other is equally demonstrable.

"If these remarks be true, the conclusion is irresistible, that the two sciences of Dentistry and Medicine, are essentially and inseparably connected, and that as a necessary consequence, the dental student, to become properly acquainted with his profession, must also combine a general knowledge of medicine, anatomy and physiology." (pp. 94, 95.)

Now, it may be laid down as an invariable principle, that no progress can be made in pathological investigations, until both these anatomical and physiological relations, are fully understood. And we may here properly inquire in what work upon dental surgery, are these *systematically* and *clearly* set forth, so as to come within the comprehension of the *dental* student? If such a work is extant, we confess we are not acquainted with it. We have many works well adapted to those already well versed in *medical* science, but none which are as well adapted to the wants both of the unread student, and him who is already familiar with first principles, as the treatise under consideration. We can but very briefly allude to the other great additions to the first edition, comprising the second and fifth parts of the second edition.

Part second is devoted to the "Physical Characteristics of the *Teeth, Gums,* Salivary Calculus, Fluids of the Mouth, Lips and Tongue," as indicative of peculiarities of constitution and susceptibility of the teeth, both in a state of health and disease.

A very great amount of interesting and instructive matter is contained in this part of the work. No acquisition can be of greater service to the dental surgeon, than an ability, on inspecting the mouth and teeth, to decide at once what are the required operations, and their succession, in order to restore it to health, and supply (when required) such teeth as are desired. To do this readily and correctly, requires a familiarity with the physical characteristics of the teeth and mouth, both in a state of health and disease, not easily obtained. Although close observation is necessary, after all that one may learn from books, yet this task may be greatly abridged by availing himself of the very numerous facts and observations which may be learned from this part of Dr. H's. work, and which are not only the result of his own laborious researches upon this subject, but include also those of the most eminent men, who have treated upon these topics.

Part fifth is devoted to the *Diseases* of the Maxillary Sinus.

Every dental practitioner of any experience, knows well the occult nature of the different diseases connected with the maxillary sinus. When we also take into consideration, the fact, that there are few diseases which have not received more attention from medical or dental writers, and that many of these diseases are the most formidable which either dental or surgical practitioners are called upon to treat, we may well approbate any work which furnishes us all the combined lights upon this important subject. No one who has ever found difficulty in the treatment of these diseases, (and there are few in extensive practice who have not,) can read this part of the work without acknowledging its great merit and ascribing to its author the credit of having contributed vastly to the resources, not only to the dental, but to the medical profession, and thereby to the meliorating the sufferings of the human race.

